# Syndromic molecular testing in mechanically ventilated patients with severe pneumonia: a supportive diagnostic approach

**DOI:** 10.1128/spectrum.02155-25

**Published:** 2025-11-21

**Authors:** Tina Uršič, Kaja Erzar, Katja Seme, Darja Keše, Matjaž Jereb, Franc Strle, Miroslav Petrovec

**Affiliations:** 1 Institute of Microbiology and Immunology, Faculty of Medicine, University of Ljubljana 37664, Ljubljana, Slovenia; 2 Department of Infectious Diseases, University Medical Centre Ljubljana 37667, Ljubljana, Slovenia; 3 Department of Infectious Diseases and Epidemiology, Faculty of Medicine, University of Ljubljana 37664, Ljubljana, Slovenia; Nationwide Children's Hospital, Columbus, Ohio, USA

**Keywords:** molecular methods, fungi, bacteria, viruses, intensive care unit, severe pneumonia

## Abstract

Rapid identification of pathogens is critical in severe pneumonia patients for timely therapy in mechanically ventilated intensive care unit patients. Conventional diagnostics are time-consuming. The Allplex Respiratory Panels 1–4 detect 16 viruses and seven bacteria and provide faster results; however, because this molecular assay does not yield viable organisms, antimicrobial susceptibility testing cannot be performed. We compared conventional diagnostics with commercial panels to evaluate diagnostic performance and clinical utility. In this prospective study, a total of 30 bronchoalveolar lavage fluids and 80 tracheal aspirates were obtained from 110 unique ICU patients with severe pneumonia (January 2014 and April 2016). Conventional diagnostics were performed during illness, whereas testing of these specimens by commercial panels was performed retrospectively. Pathogen detection rates and co-detections were analyzed by pneumonia type (community-acquired, hospital-acquired, and ventilator-associated). Conventional diagnostics detected pathogens in 87 of 110 patients (79.1%) versus 70 of 110 (63.6%) detected by the commercial approach (*P* = 0.016). Detection of viruses was most frequent, predominantly influenza A and human rhinoviruses; common bacterial detections included *Haemophilus influenzae*, *Legionella pneumophila*, and *Streptococcus pneumoniae*. Co-detections occurred in 12.7% patients by the commercial approach and in 21.8% by the conventional approach, including virus–bacterium co-detections in 10.9% and 14.5% of cases, respectively. The commercial approach missed eight bacterial and four fungal detections identified by conventional methods. Conventional diagnostics showed higher yield in hospital-acquired pneumonia (82.5% vs 50.0%; *P* = 0.004), whereas both approaches were comparable in community-acquired cases (75% vs 73.4%). Conventional diagnostics (including culture-based methods) provide broader detection and offer the ability for phenotypic antimicrobial susceptibility testing to be performed, whereas commercial panels offer speed; together, they may optimize pathogen detection and therapy in critically ill patients.

**IMPORTANCE:**

Severe pneumonia in critically ill patients remains a major clinical challenge due to its diverse etiology, rapid progression, and the need for timely, targeted therapy. This study demonstrates that conventional diagnostic approaches—combining culture and molecular tests—identify the etiology more effectively than a commercial multiplex PCR-based syndromic panel in ICU patients. While the molecular approach offers faster results, it lacks the breadth of bacterial and fungal targets and does not provide the opportunity for antimicrobial susceptibility testing. Importantly, viral pathogens—particularly influenza A and rhinoviruses—were frequently detected, underscoring their role in severe pneumonia and the relevance of viral-bacterial co-infections. This work highlights that syndromic molecular diagnostics may be valuable for rapid screening or in community-acquired pneumonia but are insufficient for hospital- or ventilator-associated pneumonia. Our findings support a complementary diagnostic strategy to optimize pneumonia management in ICU settings, improve antimicrobial stewardship, and ultimately impact clinical outcomes for patients with life-threatening respiratory infections.

## INTRODUCTION

It has been estimated that at least 200 million pneumonia cases occur in the world every year (1). In Slovenia, with a population of 2.1 million, each year between 20,000 and 30,000, patients are treated for pneumonia as outpatients, and 5,000 to 7,000 patients are hospitalized due to a severe course of the disease (2). The proportion of hospitalized patients is the highest in preschool children, in patients with underlying chronic diseases, and the proportion is particularly high among those over 80 years of age (1, 3). The fatality rate of patients with community-acquired pneumonia (CAP) requiring hospital treatment is approximately 10% and is much higher (20%–50%) in patients who need intensive care treatment (3). The fatality rate increases with the age of the patients and is especially high in those >80 years old. The occurrence of pneumonia patients fluctuates seasonally and is strongly related to the frequency of influenza (4).

According to the infection source, pneumonia can be divided into CAP, hospital-acquired pneumonia (HAP), and ventilator-associated pneumonia (VAP). The etiology of these entities differs: the most common causative agents for CAP are *Streptococcus pneumoniae*, respiratory viruses, *Haemophilus influenzae*, and atypical bacteria such as *Mycoplasma pneumoniae* and *Legionella pneumophila* (4); the most frequent causes of HAP are *Staphylococcus aureus*, *Klebsiella pneumoniae*, and *Pseudomonas aeruginosa* (5); and the most frequent causative agents of VAP are *P. aeruginosa*, *S. aureus*, *Acinetobacter* spp., and *Stenotrophomonas* spp. (5, 6).

Analysis of the etiology of 3,854 adult European patients treated for CAP from 2003 to 2014 showed that 7% of the causes were respiratory viruses, 24% were bacteria, 6% were polymicrobial infections, and 3% were other causes. In contrast, in 60% of patients, no pathogen was detected (1, 3). However, in the last decade, studies using advanced diagnostic methods have confirmed a large proportion of pneumonia associated with viral infections and substantially increased the understanding of the role of viruses as the causative agents of pneumonia (4, 7–9). There is much more information on the etiology of pneumonia for outpatients and hospitalized patients treated at usual departments than for critically ill patients treated in intensive care units (ICUs) (4, 7).

Despite advances in microbiological testing, establishing the etiology of severe pneumonia in mechanically ventilated ICU patients remains challenging. Conventional culture-based diagnostics are time-consuming and frequently yield negative results, particularly after prior antibiotic exposure. Syndromic multiplex PCR panels offer faster pathogen identification, but their performance and clinical utility have mostly been studied in less critically ill populations (10, 11). Real-world data on their application in ICU-acquired or VAP are limited (12). This study addresses this gap by directly comparing a commercially available multiplex PCR panel with comprehensive conventional diagnostic methods in a cohort of critically ill mechanically ventilated patients.

International guidelines for the management of CAP (13) and HAP/VAP (14) primarily recommend culture-based diagnostics and do not yet endorse routine use of multiplex PCR panels in ICU pneumonia. Recent expert reviews suggest that such panels may be considered as adjunctive tools, particularly for early viral detection and antimicrobial stewardship, but emphasize the lack of robust evidence from critically ill populations. By evaluating both diagnostic approaches in severe ICU pneumonia, our study provides novel evidence to inform this debate and clarify the potential and limitations of syndromic panels in this high-risk patient group.

This prospective study assesses the detection of pathogens in lower respiratory tract specimens obtained from critically ill patients with severe pneumonia using the conventional diagnostic approach (CDA) combining bacterial culture and molecular diagnostics of respiratory viruses and atypical bacteria. Furthermore, we aimed to evaluate whether a molecular approach, the Allplex molecular diagnostic approach (ADA; Allplex Respiratory Panel (RP) assays 1–4; Seegene Inc., Korea), is suitable for detecting potential pathogens of pneumonia in critically ill mechanically ventilated patients and to highlight the potential advantages and limitations of the ADA in comparison to the current CDA.

## MATERIALS AND METHODS

### Study population, inclusion criteria, definitions

The study population consisted of consecutive mechanically ventilated adult patients treated at the ICU of the Department of Infectious Diseases, Ljubljana University Medical Center, between January 2014 and April 2016 for severe CAP, HAP, or VAP.

Pneumonia cases were classified as CAP, HAP, or VAP based on established international guidelines by the Infectious Diseases Society of America, American Society of Microbiology, and American Thoracic Society (13–16). Diagnostic criteria for CAP are clinical presentation, including cough, fever, and radiologically detected lung infiltrate at the beginning of the illness in a community setting.

Diagnostic criteria for HAP are radiologically confirmed lung infiltrate that appeared >48 hours after admission to the hospital in a patient who simultaneously had at least two of the following indicators: body temperature>38.0°C, presence of leukocytosis or leukopenia, or purulent tracheobronchial discharge. Patients with HAP were either transferred to the ICU from other departments due to severe HAP or developed HAP during treatment in the ICU.

Diagnostic criteria for VAP are the formation of a new lung infiltrate or the spread of already known lung infiltrates in a patient that had been mechanically ventilated for >48 hours and had at least two of the following three symptoms: body temperature >38.0°C, presence of leukocytosis or leukopenia, or purulent tracheobronchial discharge.

### Microbiological analyses

**Fig 1:**
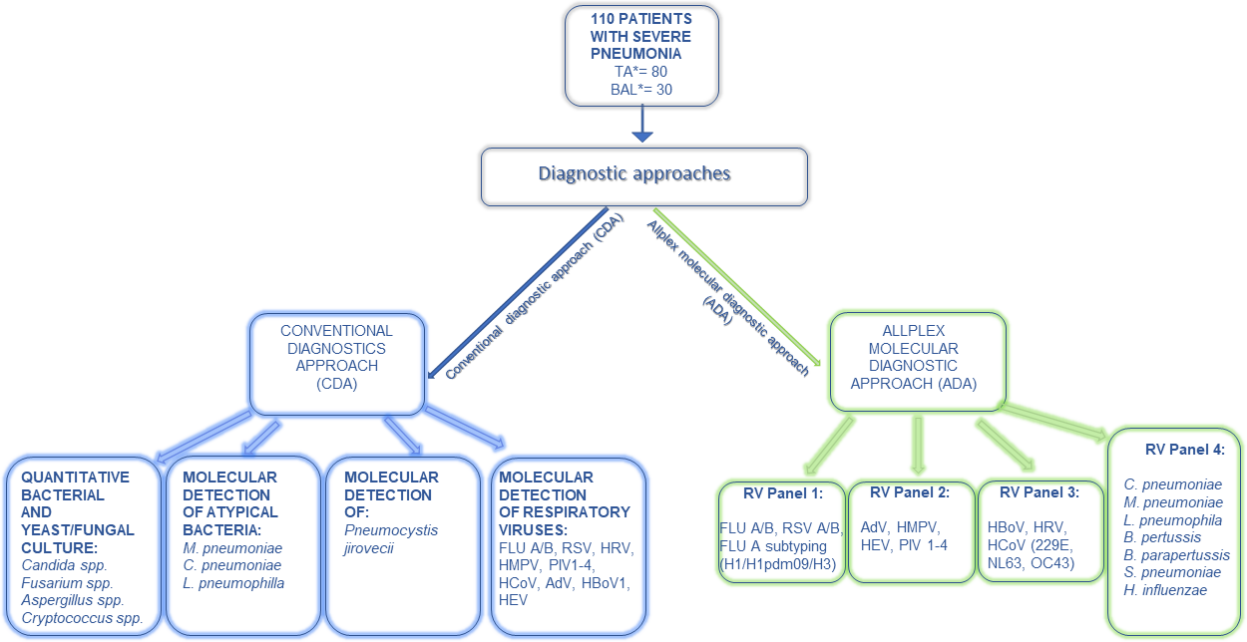
Schematic presentation of two diagnostic workflows for mechanically ventilated patients with severe pneumonia. Legend: TA*, first tracheal aspirate; BAL*, first bronchoalveolar lavage; FluA/B, influenza A/B virus; RSV, respiratory syncytial virus; HRV, human rhinovirus; HMPV, human metapneumovirus; PIV1−4, parainfluenza viruses 1–4; HCoV, seasonal coronaviruses; AdV, Adenovirus; HBoV1, human bocavirus 1; HEV, human enterovirus; *M. pneumoniae*, *Mycoplasma pneumoniae*; *C. pneumoniae*, *Chlamydia pneumoniae*; *L. pneumophila*, *Legionella pneumophila*; *B. pertussis*, *Bordetella pertussis*; *B. parapertussis*, *Bordetella parapertussis*; *S. pneumoniae*, *Streptococcus pneumoniae*; *H. influenzae*, *Haemophilus influenzae*. The left side presents methods and pathogens detected using a CDA, and the right side shows detection using the syndromic ADA.

In this study, mechanically ventilated ICU patients with pneumonia often had more than one lower respiratory tract sample (tracheal aspirate or bronchoalveolar lavage [BAL]) collected during their disease course. For consistency and to avoid repeated measures, we analyzed only the first available lower respiratory tract sample, collected at the time of pneumonia suspicion/diagnosis, using both the CDA and the ADA. The CDA testing was performed in real time while patients were experiencing ongoing clinical symptoms (2014–2016), and the same combination of CDA assays was applied to all patient samples. The ADA testing was performed retrospectively in 2018 using the same raw tracheal aspirate and BAL specimens, which had been stored at −80°C. For ADA testing, nucleic acids were re-extracted from these stored samples before analysis. The diagnostic workflow is depicted in Fig. 1.

#### Conventional diagnostic approach

Microbiologic diagnostics according to the conventional approach included quantitative bacterial culture. The quality and adequacy of respiratory specimens were assessed by microscopic examination at 100× magnification following Gram staining. Samples were considered acceptable if they contained >25 leukocytes and <10 epithelial cells per field. Samples not meeting these criteria were rejected for diagnostic processing, in line with clinical practice. Established quantitative culture thresholds to distinguish colonization/contamination from true infection were followed. For BAL specimens, ≥10⁴ CFU/mL was considered significant, and for endotracheal aspirates ≥10⁵ CFU/mL, as described previously (17–19). Matrix-assisted laser desorption/ionization time-of-flight mass spectrometry (Bruker Daltonics, Bremen, Germany) was used for microbial identification of recovered isolates. Moreover, selective mycology media including Sabouraud dextrose agar and CHROMagar *Candida* were used for the isolation and identification of *Candida* species, as well as other clinically important fungi, such as *Fusarium* spp., *Aspergillus* spp., and *Cryptococcus* spp. Additionally, direct Gram staining and India ink staining were performed for the presumptive identification of *Cryptococcus* spp., whereas direct microscopy with calcofluor white staining was used for the detection of *Aspergillus* and *Fusarium* spp. The detection of respiratory viruses—including influenza A and B (flu A/B), respiratory syncytial viruses (RSVs), adenoviruses (AdV), human bocavirus 1 (HBoV1), seasonal coronaviruses (HCoV), human metapneumoviruses (HMPV), parainfluenza viruses 1–4 (PIV 1-4), human rhinoviruses (HRV), and enteroviruses (EV)—was carried out using in-house real-time RT-PCR as described previously (20). *M. pneumoniae*, *L. pneumophila*, and *C. pneumoniae* were detected using the Chla/Myco pneumo and Legio pneumo/Cc R-GENE Kit according to the manufacturer’s instructions (Argene by Biomerieux, France) and *Pneumocystis jirovecii* using in-house real-time PCR using the primers and probe described previously (21).

#### Detection of bacteria and viruses using ADA

From BAL and TA samples, nucleic acids were retrospectively re-extracted and tested using Allplex RPs 1, 2, 3, and 4 by Seegene (Seegene Inc., Korea). RP1 was used for detecting FluA/B and RSV; RP2 for AdV, HMPV, PIV1–4, and EV; RP3 for HBoV 1–4, HRV, and seasonal HCoV; and RP4 for bacterial pathogens: *Chlamydia pneumoniae*, *M. pneumoniae*, *L. pneumophila*, *Bordetella pertussis*, *Bordetella parapertussis*, *S. pneumoniae*, and *H. influenzae* (Fig. 1). Extraction of nucleic acids was performed using the STARMag 96 × 4 Universal Cartridge Kit (Seegene Inc., Korea) using a Microlab NIMBUS instrument, and real-time RT-PCR was performed using the CFX96 real-time PCR system (Bio-Rad, USA). If there was an insufficient patient sample for nucleic acid extraction and subsequent ADA testing, the patient was excluded from the study. During conventional diagnostic analysis, there were no failed sample extractions and no invalid results. In the ADA testing, three samples initially yielded invalid results; these were successfully re-extracted, and ADA was subsequently completed successfully. Syndromic molecular/PCR testing was performed and interpreted blinded to CDA, with data compared only after all analyses were completed to minimize interpretation bias.

### Statistics

Continuous variables were summarized using medians and (interquartile) ranges. Student’s *t*-test was used for continuous variable calculation of the statistical significance (mean Ct values), and categorical variables were presented as frequencies with percentages. Fisher’s exact test was used for calculating the statistical significance of categorical variables.

## RESULTS

Basic characteristics of patients with severe pneumonia are presented in Table 1.

**TABLE 1:** Basic information on 110 mechanically ventilated patients treated for severe pneumonia^*a*^

Patient characteristics	Patients, *n* (%)
Sex	
Women	31 (29.2)
Men	79 (70.8)
Age median (years)	67 (IQR 57–78)
Sample type	
Tracheal aspirate	80 (72.7)
BAL	30 (27.3)
Pneumonia type	
CAP	64 (58.2)
HAP	40 (36.4)
VAP	6 (5.4)

^*a*^ IQR, interquartile range.

A comparison of the CDA and ADA approaches in terms of pathogen detection rates, including co-detections, is presented in Table 2. The CDA showed a higher percentage of respiratory pathogens detected (87/110, 79.1% vs 70/110, 63.6%; *P* = 0.016). The difference arises from the limited number of bacterial and fungal targets in ADA.

**TABLE 2:** Comparison of pathogen detection in TA and BAL samples using CDA and ADA

Characteristics	CDA	ADA
	Patients, *n* (%)	Patients, *n* (%)
Total	110 (100)	110 (100)
NEGATIVE samples	23 (20.9)	40 (36.4)
POSITIVE samples	87 (79.1)	70 (63.6)
Single pathogen detected	63 (57.3)	56 (50.9)
Single viral pathogens	34 (31.0)	39 (35.4)
Influenza A virus	15 (13.6)	16 (14.5)
HRV	10 (9.1)	12 (10.9)
RSV	3 (2.7)	4 (3.6)
HMPV	2 (1.8)	2 (1.8)
PIV 1–4	2 (1.8)	2 (1.8)
Human coronaviruses	1 (0.9)	1 (0.9)
Influenza B virus	1 (0.9)	1 (0.9)
Adenovirus	0	1 (0.9)
Single bacterial pathogens	25 (22.7)	17 (15.4)
*S. pneumoniae*	3 (2.7)	8 (7.3)
*H. influenzae*	4 (3.6)	5 (4.5)
*L. pneumophila*	4 (3.6)	4 (3.6)
MRSA	3 (2.7)	NA^*a*^
*P. aeruginosa*	2 (1.8)	NA
*S. aureus*	1 (0.9)	NA
*S. marcescens*	2 (1.8)	NA
*S. maltophilia*	1 (0.9)	NA
*Escherichia coli*	1 (0.9)	NA
*K. pneumoniae*	1 (0.9)	NA
Extended-spectrum *K. pneumoniae* (ESBL)	1 (0.9)	NA
*Acinetobacter baumannii*	1 (0.9)	NA
*C. freundii*	1 (0.9)	NA
*M. pneumoniae*	0	0
*C. pneumoniae*	0	0
*B. pertussis/B. parapertussis*	0	0
Single yeast pathogens	4 (3.6)	-
*P. jirovecii*	3 (2.7)	NA
*C. albicans*	1 (0.9)	NA
Co-detections	24 (21.8)	14 (12.7)
Virus–bacterium	16 (14.5)	12 (10.9)
Influenza A virus/*S. aureus*	2 (1.8)	0
Influenza A virus/*H. influenzae*	1 (0.9)	2 (1.8)
Influenza A virus/*S. pneumoniae*	1 (0.9)	2 (1.8)
Influenza A virus/*E. faecalis*	2 (1.8)	0
Influenza A virus/HRV/*K. pneumoniae*/*S. pneumoniae*	1 (0.9)	0
Influenza A virus/*S. pneumoniae*/*H. influenzae*	0	2 (1.8)
Influenza B virus/*Streptococcus equi*	1 (0.9)	0
Influenza B virus /*S. aureus*	1 (0.9)	0
RSV/*Escherichia coli*	1 (0.9)	0
RSV/*S. pneumoniae*	2 (1.8)	0
RSV/*H. influenzae*	0	2 (1.8)
HMPV/*S. pneumoniae*	2 (1.8)	1 (0.9)
Human coronavirus/adenovirus/*S. aureus*	1 (0.9)	0
HRV/*S. pneumoniae*	0	2 (1.8)
HRV/*P. aeruginosa*/*C. freundii*	1 (0.9)	0
HRV/*S. pneumoniae*/*H. influenzae*	0	1 (0.9)
Bacterium–bacterium	6 (5.5)	1 (0.9)
*H. influenzae***/***C. propinquum*	1 (0.9)	0
*P. aeruginosa***/***E. faecalis*	1 (0.9)	0
*S. pneumoniae***/***H. influenzae*	1 (0.9)	1 (0.9)
*S. aureus***/***P. aeruginosa*	1 (0.9)	0
*K. pneumoniae***/***S. maltophilia*	1 (0.9)	0
*P. aeruginosa***/***Escherichia coli*	1 (0.9)	0
Virus–virus		
HMPV/adenovirus	1 (0.9)	1 (0.9)
Virus–fungus		
HRV/*P. jirovecii*	1 (0.9)	NA

^*a*^ NA, not applicable.

### Seasonal pattern of viral pneumonia

**Fig 2:**
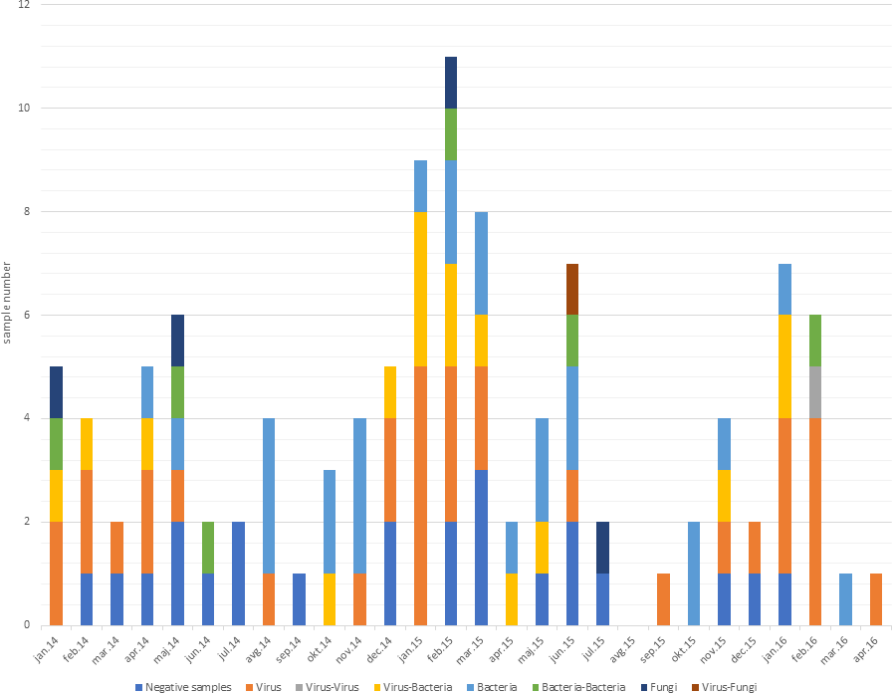
Seasonality pattern of severe pneumonia cases. Legend: samples of severe pneumonia patients during the study period are colored depending on the type of pathogen detected, single or co-detections.

Because respiratory viruses tend to follow seasonal patterns, a higher incidence of viral pneumonia was observed in the winter months. This was observed for each season during the study period, from January 2014 to April 2016 (Fig. 2).

### Single infections and co-detections in mechanically ventilated patients with severe pneumonia

**Fig 3:**
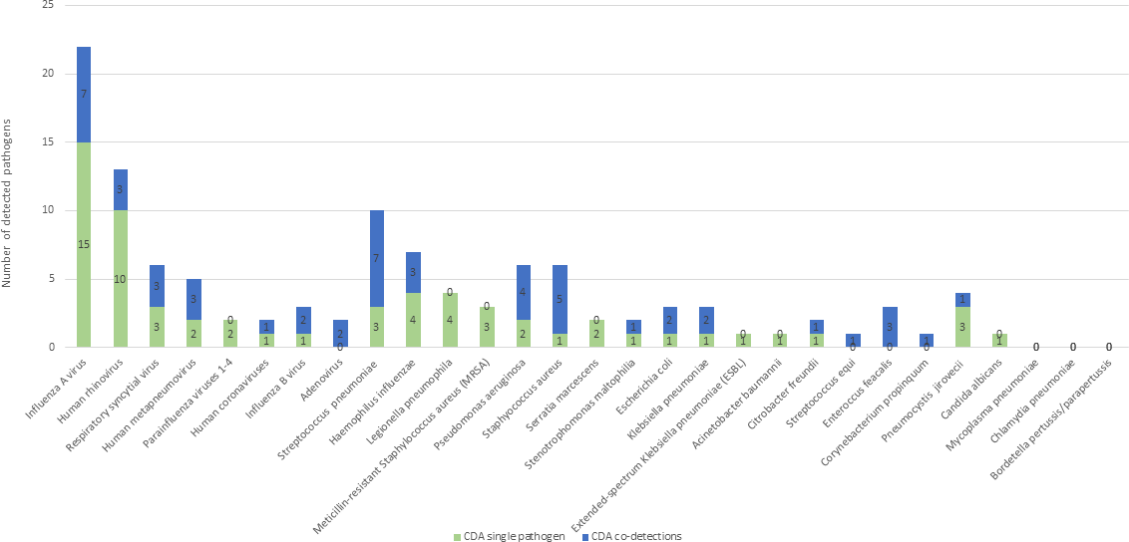
Number of pathogens detected using the CDA, including numbers of single and co-detections. Legend: The figure shows pathogen detection (single or co-detections) using the conventional approach.

**Fig 4:**
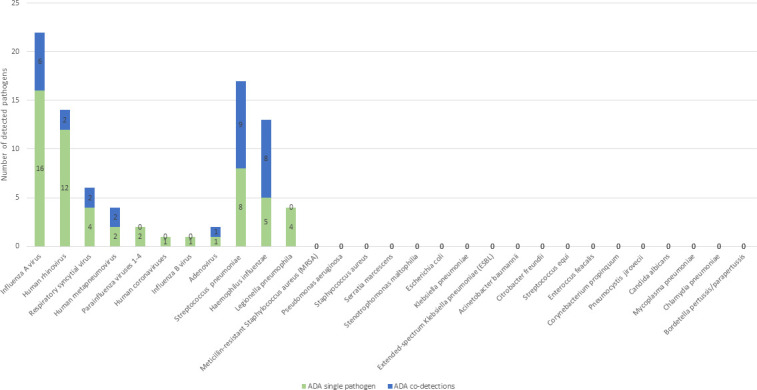
Number of pathogens detected using Allplex Seegene RPs 1–4, including numbers of single and co-detection by pathogen. Legend: The figure presents pathogens detected (single or co-detections) using the Allplex Seegene molecular diagnostic approach.

Combined results, using both the CDA and the ADA, showed that most frequently detected viruses were Flu A and HRV, and the most frequently detected bacteria were *H. influenzae*, *L. pneumophila*, and *S. pneumoniae. H. influenzae* and *S. pneumoniae* were also most frequently seen in co-detections (Fig. 3 and 4).

**Fig 5:**
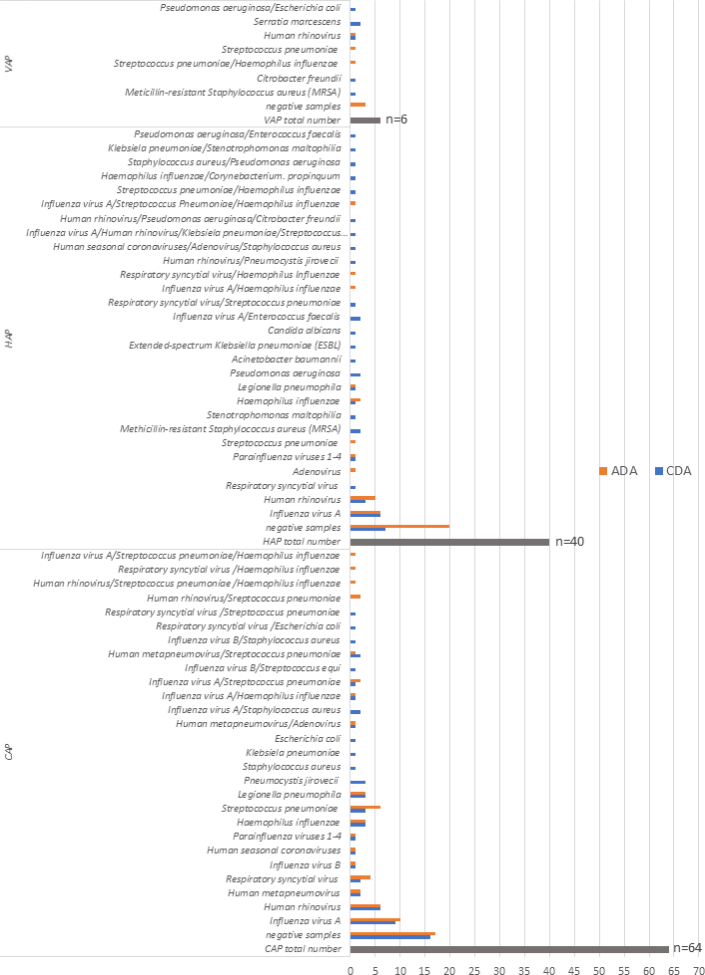
Patients with severe pneumonia and pathogens detected by pneumonia type. Legend: CAP, community-acquired pneumonia; HAP, hospital-acquired pneumonia; VAP, ventilator-associated pneumonia. Blue bars represent pathogens detected using the classical diagnostic approach, and orange bars pathogens detected using the ADA.

Comparison of the two approaches revealed that the ADA missed 14 (12.7%) bacterial single infections and four (3.6%) fungal infections (three *P. jirovecii* [Ct values: 23.2, 25.4, 29.4]) and one *Candida albicans* (>10^5^ CFU/mL), as well as 12 (10.9%) co-detections detected using the CDA. However, additional viral single infections were detected by ADA in five (4.5%) in comparison to the current CDA (Table 2 and Fig. 5). Regarding bacteria, using the CDA, *S. pneumoniae* was detected in 10 patients, whereas using ADA, it was detected in 17 patients (Fig. 3 and 4). The mean Ct value for *S. pneumoniae* detected by ADA was significantly lower in samples that were positive by both CDA and ADA compared to those positive only by ADA (22.81 vs 28.89; *P* = 0.001). A similar pattern was seen for *H. influenzae* detection. It was detected in seven patients using the CDA and in 13 patients using ADA. The mean Ct value for *H. influenzae* detected by ADA was significantly lower in samples that were positive by both CDA and ADA, compared to those positive only by ADA (21.97 vs 30.53; *P* < 0.001). Five of seven pneumonia patients who were negative by CDA but positive for *S. pneumoniae* by ADA and all six patients who were negative by CDA but positive for *H. influenzae* by ADA had already received antibiotic treatment before sample collection.

Using the existing CDA, five multi-resistant bacteria, four *S*. *aureus* (MRSA), and one case of *K. pneumoniae* (extended-spectrum beta-lactamase [ESBL]) were detected; all were missed using the ADA due to a lack of associated targets for these organisms.

### Detected pathogens by pneumonia type

Of 110 patients, 64 had severe CAP. Using the CDA, a single virus was detected in 22 patients (34.4%) with Flu A (*n* = 9) and HRV (*n* = 6), accounting for 15/22 (23.4%) of these single virus infections. Single bacterial infections were detected in 12 (18.7%) patients, with *S. pneumoniae* (*n* = 3), *H. influenzae* (*n* = 3), and *L. pneumophila* (*n* = 3) accounting for the majority of single bacterial infections (Table 3). Three patients (4.7%) with CAP, with three single fungal infections caused by *P. jirovecii*, were detected (Fig. 5). Of the 11 cases in which several pathogens were detected together, a virus and a bacterium occurred together in 10 cases, with more than half of these cases containing combinations of Flu A/B virus, HMPV, or RSV with *S. pneumoniae*, *H. influenzae*, *or S. aureus* (Fig. 5).

**TABLE 3:** Pathogen detection in patients according to pneumonia type^*a*^

Patient characteristics	CAP	HAP	VAP
Total number of patients; *N* = 110 (%)	64 (58.2)	40 (36.4)	6 (5.4)
Single pathogen detected by CDA	37/64 (57.8)	21 (52.5)	5 (16.7)
Virus	22 (34.4)	11 (27.5%)	1 (16.7)
Bacterium	12 (18.7)	9 (22.5)	4 (66.7)
Fungus	3 (4.7)	1 (2.5)	0
Co-detections detected by CDA	11 (17.2)	12 (30.0)	1 (16.7)
Virus–bacterium	10 (15.6)	6 (15.0)	0
Bacterium–bacterium	0	5 (12.5)	1 (16.7)
Virus–virus	1 (1.6)	0	0
Virus–fungus	0	1 (2.5)	0
Positive patients by CDA	48/64 (75.0)	33/40 (82.5)	6/6 (100)
Single pathogen detected by ADA	37/64 (57.8)	17/40 (42.5)	2/6 (33.3)
Virus	25 (39.1)	13 (32.5)	1 (16.7)
Bacterium	12 (18.8)	4 (10.0)	1 (16.7)
Fungus	NA^*b*^	NA	NA
Co-detections detected by ADA	10 (15.6)	3 (7.5)	1 (16.7)
Virus–bacterium	9 (14.1)	3 (7.5)	0
Bacterium–bacterium	0	0	1 (16.7)
Virus–virus	1 (1.5)	0	0
Positive patients by ADA	47/64 (73.4)	20/40 (50.0)	3/6 (50.0)

^*a*^ ADA, Allplex molecular diagnostic approach; CAP, community-acquired pneumonia; CDA, conventional diagnostic approach; HAP, hospital-acquired pneumonia; VAP, ventilator-associated pneumonia.

^*b*^ NA, not applicable.

Using the ADA, single virus infections were found in 25 (39.0%) patients with Flu A (*n* = 10) and HRV (*n* = 6), corresponding to 16 (25.0%) of the single viral infections. Single bacterial infections were again identified in 12 (18.8%) patients, with *S. pneumoniae* (*n* = 6), *H. influenzae* (*n* = 3), and *L. pneumophila* (*n* = 3) accounting for all 12 single bacterial infections. In contrast, more than one pathogen was detected in 10 patients (15.6%), with nine cases involving a combination of viruses and bacteria. Flu A and HRV were most frequently detected together with *S. pneumoniae* and *H. influenzae*. Overall, the two approaches were comparable in identifying the potential cause of CAP (48/64, 75% vs 47/64, 73.4% for the CDA and the ADA, respectively).

In 40 patients with severe HAP, the CDA identified single viral infections in 11 patients (27.5%), with Flu A (*n* = 6) and HRV (*n* = 3) accounting for 9 (22.5%) of these cases (Table 3). Single bacterial infections were detected in nine patients (22.5%), most frequently methicillin-resistant *S. aureus* (MRSA) (*n* = 2) and *P. aeruginosa* (*n* = 2), which together accounted for 4 (10 %) single bacterial infections. A single fungal infection with *C. albicans* was detected in one patient (2.5%; Fig. 5). Twelve patients (30.0%) had multiple pathogens, including six virus–bacterium combinations and five bacterium–bacterium combinations. The most common virus–bacterium combinations were Flu A or HRV with *S. pneumoniae* or *Enterococcus faecalis*. Bacterium–bacterium combinations included *H. influenzae*, *P. aeruginosa*, *S. aureus*, *K. pneumoniae*, *Stenotrophomonas maltophilia*, *Citrobacter freundii*, and *Corynebacterium propinquum*. In addition, virus–fungus co-detection (HRV and *P. jirovecii*) was observed.

Using the ADA, single viral infections were detected in 13 patients (32.5%), with Flu A (*n* = 6) and HRV (*n* = 5) accounting for 11 (27.5%) of these cases. Single bacterial infections were detected in four patients (10.0%), including *H. influenzae* (*n* = 2), *S. pneumoniae* (*n* = 1), and *L. pneumophila* (*n* = 1; Table 3). Virus–bacterium combinations were detected in three patients (7.5%), with *H. influenzae* present in all cases and Flu A in two cases.

Overall, Flu A and HRV were the most frequently detected viruses in both approaches. In CDA, the most common bacteria were MRSA and *P. aeruginosa*, whereas *H. influenzae* was predominant in the ADA. The etiology of HAP was more commonly identified with CDA than with the ADA (33/40, 82.5% vs 20/40, 50.0%; *P =* 0.004) (Table 3 and Fig. 5). Six patients had severe VAP (Fig. 5). The CDA detected a single viral infection (HRV) in one patient, single bacterial infections in four patients (*Serratia marcescens n* = 2, *C. freundii n* = 1, and MRSA *n* = 1), and a bacterium–bacterium combination (*P. aeruginosa* and *E. coli*) in one patient. The etiological pathogen was thus identified in all six patients (100%). With the ADA, pathogens were only detected in three patients (50%): in one with HRV, in one with *S. pneumoniae*, and in one with a joint detection of *S. pneumoniae* and *H. influenzae* (Fig. 5; *P =* 0.1818).

## DISCUSSION

Although modern diagnostic methods have considerably improved the etiological detection of microbial infections, pneumonia diagnosis remains challenging due to the wide range of potential pathogens and difficulties in obtaining high-quality samples. Combining classical bacteriology, serology, antigenic, and molecular testing allows pathogen identification in 38%–76% of pneumonia cases (8, 9, 22–24), which is not optimal. Furthermore, such an approach is labor-intensive and time-consuming, often requiring days for definitive results. While new diagnostic approaches are needed, they should be critically assessed against conventional methods (25).

Several commercial multiplex molecular RPs are now available for detecting respiratory pathogens (25–28).

This study evaluated the Allplex Seegene RP 1–4 kits (ADA) and compared the results with our CDA in severely ill mechanically ventilated ICU patients with pneumonia. To our knowledge, the ADA, which detects 16 viral and seven bacterial pathogens, has not been evaluated in such a patient group.

Our CDA identified a potential etiologic pathogen in 79.1% of cases, supporting the value of combining molecular and conventional methods, which may improve detection by 20%–50% (29, 30). Testing the same specimens, the ADA identified the causative agent(s) in 63.6% of cases within 6 hours. Despite the faster turnaround, 15.5% of cases diagnosed by the CDA remained undetected using the ADA due to its limited bacterial range. Specifically, the ADA does not include several common HAP/VAP pathogens such as *S. aureus*, *K. pneumoniae*, other *Enterobacteriaceae*, and *P. aeruginosa*, nor does it include fungal pathogens (1, 8, 9, 22, 29). As a result, several bacterial infections (including five multidrug-resistant bacteria) and four fungal infections, detected using the currently used CDA, were missed by the ADA. Etiological diagnosis is important for appropriate treatment in general and may be critical in the management of patients with severe pneumonia in the ICU (31, 32).

Using the CDA combined with molecular testing, we identified viral pathogens in 47.3% of cases, with single-virus infections in 31.0%. As expected, seasonal occurrence of viral pneumonia was observed (Fig. 2) (7, 22–24, 29, 33). Influenza A virus was the most common viral cause, accounting for 20% of pneumonia cases. Bacteria were found in 42.7% of cases, with single bacterial infections in 22.7%. *S. pneumoniae* and *H. influenzae* were detected most frequently (9.1% and 6.4%, respectively), consistent with previous reports (3, 32, 34, 35). In CAP, bacteria usually account for more than half of the agents identified, although prevalence varies widely (11%–77%) (22–24, 29, 36).

We identified four (3.6%) *L. pneumophila* cases, all confirmed by urine antigen. We also confirmed three cases of pneumocystis pneumonia in immunocompromised patients with a high fungal burden in a clinical context (two patients after kidney transplantation and one patient with non-Hodgkin’s lymphoma). No atypical bacteria, such as *C. pneumoniae* and *M. pneumoniae*, were detected, although they are reported in 3%–15% of CAP (22–24, 29, 33); however, these bacteria are uncommon causes of severe pneumonia. Furthermore, their absence in this study may also be due to our older ICU cohort (median age 67 years). A 10-year Slovenian study reported cyclic *M. pneumoniae* epidemics every 3–7 years, primarily affecting children and younger adults, with lower prevalence in those over 40 (37–39).

Acute lower respiratory tract infections are often (in 10%–56% of patients) associated with the presence of more than one agent (22–24, 29, 33, 40). In our study, co-detections were found in 21.8% of patients, with virus–bacterium combinations most commonly detected (14.5%). The main viral players in co-infections were influenza A, rhinoviruses, and RSV, whereas *S. pneumoniae* and *H. influenzae* were the most common among bacteria, which is in line with a previous report (29). An elegant explanation for virus–bacteria combinations is that having previous viral lower respiratory infections increases susceptibility to bacterial superinfections (7, 40, 41).

The results of this study indicate that the ADA is most suitable for the diagnostics of CAP because by using this approach, we were able to detect etiology in all but one out of 64 cases in which the etiology was demonstrated using the CDA. However, the system is less well designed for HAP. Due to the limited range of pathogens that can be detected using the ADA, the etiologic cause was confirmed significantly less often in comparison to our CDA (20/40, 50% vs 33/40, 82.5%; *P* = 0.008). A similar trend was also valid for VAP (3/6, 50% vs 6/6, 100%; *P* = 0.182). On the other hand, molecular tests detected the presence of bacteria more often than bacterial culture. In our study, bacterial culture was positive in only 58.8% of *S. pneumoniae* cases and 53.8% of *H. influenzae* cases with a positive Allplex molecular result, respectively. This discrepancy probably reflects the limited sensitivity of the culture, but previous antibiotic treatment may also have reduced the viability of the bacteria and thus the yield of the culture. Detection of bacteria by conventional culture in ICU patients is particularly difficult due to the low bacterial load and frequent prior antibiotic exposure. Antibiotics were administered to 5/7 patients (71.4%) that were positive for *S. pneumoniae* by the ADA but negative by the CDA and to all six patients (100%) that were positive for *H. influenzae* by the ADA but negative by the CDA, respectively. Similarly, 11/17 (64.7%) and 10/13 (76.9%) patients positive for *S. pneumoniae* and *H. influenzae* by the ADA, respectively, had received antibiotics before sampling. These data suggest that prior antibiotic exposure may have disproportionately reduced the sensitivity of the cultures, biasing the comparison in favor of the ADA. Nevertheless, conventional culture remains essential because it allows for isolate recovery essential for antimicrobial susceptibility testing performance, currently irreplaceable by molecular assays. (15).

Two Conformité Européenne-marked PCR-based tests are Food and Drug Administration-approved for pneumonia diagnosis: the BioFire FilmArray Pneumonia Panel (Biomereux, France), which detects 18 bacteria (including three atypical ones), seven resistance genes, and eight viruses in 90 minutes (27, 42), and the Unyvero Pneumonia Panel (Curetis, Germany), which detects 21 bacteria and one fungal pathogen semi-quantitatively and identifies 15 resistance genes in approximately 5 hours. Both have been evaluated in various settings (42–45) but were unavailable during our study.

In conclusion, in our study on mechanically ventilated patients treated for severe pneumonia in the ICU, our CDA identified a potential pathogen in 79.1% of cases, whereas testing the same specimens using the ADA detected the causative agent(s) in 63.6% of cases. This commercial molecular diagnostic approach proved to be very good in the detection of etiologic pathogens in the subgroup of patients with CAP, but it was less useful in the detection of etiologic pathogens in patients with HAP or VAP due to the limited range of bacterial targets*.* Consequently, in mechanically ventilated patients treated for severe pneumonia in the ICU, the ADA may be best utilized for patients with CAP or due to a short turnaround time (6 hours) as a rapid screening method, which can provide basic information on the same day. The ADA platform was selected as the test comparator in our study because it was the most cost-effective molecular RP available in our hospital. Alternative platforms, such as the Unyvero Pneumonia panel, were prohibitively expensive for evaluation, and the BioFire FilmArray Pneumonia panel was only introduced to the market at the end of 2018. Despite its limited pathogen coverage, the ADA allowed timely results to guide clinical decision-making regarding antibiotic therapy, which was a key priority in our study.

Future studies should explore the clinical interpretation of Ct values in molecular testing, their correlation with culture standards, and how molecular diagnostics could integrate with traditional microbiology to optimize treatment strategies (46). Expanding the range of detectable pathogens in commercial molecular panels, incorporating resistance gene detection, and further shortening turnaround times could strengthen the role of molecular diagnostics and therefore further improve pneumonia management as well as guide antimicrobial therapy more precisely (42).

Recently, metagenomic next-generation sequencing has emerged as a promising tool for pneumonia diagnostics. Its high-throughput capacity and sensitivity allow for improved pathogen identification, particularly in severe pneumonia cases for which conventional methods may be limited (46).

The findings of this study exposed the complementary nature of molecular and CDAs. Molecular methods provide rapid and broad pathogen detection, but conventional techniques remain essential for bacterial culturing and antimicrobial resistance testing. In our hospital setting, a hybrid diagnostic model is implemented by performing the ADA on respiratory specimens at the time of collection, in parallel with conventional quantitative bacterial cultures. Molecular results provide early identification of viral, atypical, and common bacterial pathogens to guide timely antimicrobial therapy, and conventional cultures allow confirmation of bacterial etiology and full susceptibility testing, which is particularly important for critically ill patients. Integration of both results through the laboratory/hospital information system enables ICU clinicians to make informed therapeutic decisions, combining speed and precision in the management of severe pneumonia.

This study has several limitations. First, it was conducted at a single tertiary care center with a regional ICU cohort, which may limit the generalizability of the findings to other settings or patient populations. Second, ADA testing was performed retrospectively and was not available to guide clinical management in real time, which may have influenced both diagnostic yield and interpretation. Third, the study lacked randomization and prospective enrollment, introducing potential selection bias. Fourth, due to its observational design, detailed patient-level outcomes—such as the impact of diagnostic findings on antimicrobial therapy adjustments, length of stay, or mortality—could not be systematically assessed. Finally, although the ADA and CDA approaches were compared using standardized workflows, variability in sample quality, timing of collection, and prior antimicrobial exposure could have affected detection rates.

## Data Availability

The data sets generated and/or analyzed during the current study are available from the corresponding author upon reasonable request.

## SUPPLEMENTAL MATERIAL

Table S1 spectrum.02155-25-s0001.docx Ct values for *S. pneumoniae* and H. influenzae upon ADA.

ASM does not own the copyrights to Supplemental Material that may be linked to, or accessed through, an article. The authors have granted ASM a non-exclusive, world-wide license to publish the Supplemental Material files. Please contact the corresponding author directly for reuse.

## References

[1] Torres A, Cilloniz C, Niederman MS, Menéndez R, Chalmers JD, Wunderink RG, van der Poll T. 2021. Pneumonia. Nat Rev Dis Primers 7:25. doi:10.1038/s41572-021-00259-0 33833230

[2] Lejko-Zupanc T, Pokorn M. 2015. Pljučnica. In Tomažič J, Strle F (ed), Tiskarna Povše: Združenje za Infektologijo, Slovensko zdravniško društvo, 1th ed

[3] Prina E, Ranzani OT, Torres A. 2015. Community-acquired pneumonia. Lancet 386:1097–1108. doi:10.1016/S0140-6736(15)60733-4 26277247 PMC7173092

[4] Alimi Y, Lim WS, Lansbury L, Leonardi-Bee J, Nguyen-Van-Tam JS. 2017. Systematic review of respiratory viral pathogens identified in adults with community-acquired pneumonia in Europe. J Clin Virol 95:26–35. doi:10.1016/j.jcv.2017.07.019 28837859 PMC7185624

[5] Jones RN. 2010. Microbial etiologies of hospital-acquired bacterial pneumonia and ventilator-associated bacterial pneumonia. Clin Infect Dis 51 Suppl 1:S81–S87. doi:10.1086/653053 20597676

[6] Huang Y, Jiao Y, Zhang J, Xu J, Cheng Q, Li Y, Liang S, Li H, Gong J, Zhu Y, Song L, Rong Z, Liu B, Jie Z, Sun S, Li P, Wang G, Qu J, Infection Assembly of Shanghai Respiratory Society. 2018. Microbial etiology and prognostic factors of ventilator-associated pneumonia: a multicenter retrospective study in Shanghai. Clin Infect Dis 67:S146–S152. doi:10.1093/cid/ciy686 30423049

[7] Ruuskanen O, Lahti E, Jennings LC, Murdoch DR. 2011. Viral pneumonia. Lancet 377:1264–1275. doi:10.1016/S0140-6736(10)61459-6 21435708 PMC7138033

[8] Johansson N, Kalin M, Tiveljung-Lindell A, Giske CG, Hedlund J. 2010. Etiology of community-acquired pneumonia: increased microbiological yield with new diagnostic methods. Clin Infect Dis 50:202–209. doi:10.1086/648678 20014950 PMC7107844

[9] Torres A, Lee N, Cilloniz C, Vila J, Van der Eerden M. 2016. Laboratory diagnosis of pneumonia in the molecular age. Eur Respir J 48:1764–1778. doi:10.1183/13993003.01144-2016 27811073

[10] Markussen DL, Serigstad S, Ritz C, Knoop ST, Ebbesen MH, Faurholt-Jepsen D, Heggelund L, van Werkhoven CH, Clark TW, Bjørneklett RO, Kommedal Ø, Ulvestad E, Grewal HMS. 2024. Diagnostic stewardship in community-acquired pneumonia with syndromic molecular testing: a randomized clinical trial. JAMA Netw Open 7:e240830. doi:10.1001/jamanetworkopen.2024.0830 38446481 PMC10918504

[11] Abelenda-Alonso G, Calatayud L, Rombauts A, Meije Y, Oriol I, Sopena N, Padullés A, Niubó J, Duarte A, Llaberia J, Aranda J, Gudiol C, Satorra P, Tebé C, Ardanuy C, Carratalà J. 2024. Multiplex real-time PCR in non-invasive respiratory samples to reduce antibiotic use in community-acquired pneumonia: a randomised trial. Nat Commun 15:7098. doi:10.1038/s41467-024-51547-8 39154071 PMC11330507

[12] Riaño-Sánchez LF, Alvarez-Moreno CA, Godoy M, Sierra CR, Castañeda MI, Cortés JA. 2025. Multiplex PCR pneumonia panel in critically ill patients did not modify mortality: a cohort study. Antibiotics (Basel) 14:245. doi:10.3390/antibiotics14030245 40149056 PMC11939521

[13] Metlay JP, Waterer GW, Long AC, Anzueto A, Brozek J, Crothers K, Cooley LA, Dean NC, Fine MJ, Flanders SA, Griffin MR, Metersky ML, Musher DM, Restrepo MI, Whitney CG. 2019. Diagnosis and treatment of adults with community-acquired pneumonia. An official clinical practice guideline of the American thoracic society and infectious diseases society of America. Am J Respir Crit Care Med 200:e45–e67. doi:10.1164/rccm.201908-1581ST 31573350 PMC6812437

[14] Kalil AC, Metersky ML, Klompas M, Muscedere J, Sweeney DA, Palmer LB, Napolitano LM, O’Grady NP, Bartlett JG, Carratalà J, El Solh AA, Ewig S, Fey PD, File TM Jr, Restrepo MI, Roberts JA, Waterer GW, Cruse P, Knight SL, Brozek JL. 2016. Management of adults with hospital-acquired and ventilator-associated pneumonia: 2016 clinical practice guidelines by the infectious diseases society of America and the American thoracic society. Clin Infect Dis 63:e61–e111. doi:10.1093/cid/ciw353 27418577 PMC4981759

[15] Miller JM, Binnicker MJ, Campbell S, Carroll KC, Chapin KC, Gilligan PH, Gonzalez MD, Jerris RC, Kehl SC, Patel R, Pritt BS, Richter SS, Robinson-Dunn B, Schwartzman JD, Snyder JW, Telford S III, Theel ES, Thomson RB Jr, Weinstein MP, Yao JD. 2018. A guide to utilization of the microbiology laboratory for diagnosis of infectious diseases: 2018 update by the infectious diseases society of America and the American society for microbiology. Clin Infect Dis 67:e1–e94. doi:10.1093/cid/ciy381 29955859 PMC7108105

[16] Miller JM, Binnicker MJ, Campbell S, Carroll KC, Chapin KC, Gonzalez MD, Harrington A, Jerris RC, Kehl SC, Leal SM Jr, Patel R, Pritt BS, Richter SS, Robinson-Dunn B, Snyder JW, Telford S 3rd, Theel ES, Thomson RB Jr, Weinstein MP, Yao JD. 2024. Guide to utilization of the microbiology laboratory for diagnosis of infectious diseases: 2024 update by the Infectious Diseases Society of America (IDSA) and the American Society for Microbiology (ASM). Clin Infect Dis:ciae104. doi:10.1093/cid/ciae104 38442248

[17] Vallés J, Rello J, Fernández R, Blanch L, Baigorri F, Mestre J, Matas L, Marín A, Artigas A. 1994. Role of bronchoalveolar lavage in mechanically ventilated patients with suspected pneumonia. Eur J Clin Microbiol Infect Dis 13:549–558. doi:10.1007/BF01971305 7805682

[18] Miller PR, Meredith JW, Chang MC. 2003. Optimal threshold for diagnosis of ventilator-associated pneumonia using bronchoalveolar lavage. J Trauma 55:263–267. doi:10.1097/01.TA.0000075786.19301.91 12913635

[19] Baselski V, Klutts JS, Baselski V, Klutts JS. 2013. Quantitative cultures of bronchoscopically obtained specimens should be performed for optimal management of ventilator-associated pneumonia. J Clin Microbiol 51:740–744. doi:10.1128/JCM.03383-12 23284021 PMC3592072

[20] Uršič T, Miksić NG, Lusa L, Strle F, Petrovec M. 2016. Viral respiratory infections in a nursing home: a six-month prospective study. BMC Infect Dis 16:637. doi:10.1186/s12879-016-1962-8 27814689 PMC5097393

[21] Alanio A, Desoubeaux G, Sarfati C, Hamane S, Bergeron A, Azoulay E, Molina JM, Derouin F, Menotti J. 2011. Real-time PCR assay-based strategy for differentiation between active Pneumocystis jirovecii pneumonia and colonization in immunocompromised patients. Clin Microbiol Infect 17:1531–1537. doi:10.1111/j.1469-0691.2010.03400.x 20946413

[22] Morgan AJ, Glossop AJ. 2016. Severe community-acquired pneumonia. BJA Educ 16:167–172. doi:10.1093/bjaed/mkv052 32288942 PMC7104960

[23] Bjarnason A, Westin J, Lindh M, Andersson L-M, Kristinsson KG, Löve A, Baldursson O, Gottfredsson M. 2018. Incidence, etiology, and outcomes of community-acquired pneumonia: a population-based study. Open Forum Infect Dis 5:ofy010. doi:10.1093/ofid/ofy010 29479548 PMC5804852

[24] Jain S, Self WH, Wunderink RG, Fakhran S, Balk R, Bramley AM, Reed C, Grijalva CG, Anderson EJ, Courtney DM, et al.. 2015. Community-acquired pneumonia requiring hospitalization among U.S. adults. N Engl J Med 373:415–427. doi:10.1056/NEJMoa1500245 26172429 PMC4728150

[25] Poole S, Clark TW. 2020. Rapid syndromic molecular testing in pneumonia: the current landscape and future potential. J Infect 80:1–7. doi:10.1016/j.jinf.2019.11.021 31809764 PMC7132381

[26] Stafylaki D, Maraki S, Vaporidi K, Georgopoulos D, Kontoyiannis DP, Kofteridis DP, Chamilos G. 2022. Impact of molecular syndromic diagnosis of severe pneumonia in the management of critically ill patients. Microbiol Spectr 10:e0161622. doi:10.1128/spectrum.01616-22 36154180 PMC9603977

[27] Ferrer J, Clari MÁ, Giménez E, Carbonell N, Torres I, Blasco ML, Albert E, Navarro D. 2023. The Biofire Filmarray Pneumonia Plus panel for management of lower respiratory tract infection in mechanically-ventilated patients in the COVID-19 era: a diagnostic and cost-benefit evaluation. Diagn Microbiol Infect Dis 105:115847. doi:10.1016/j.diagmicrobio.2022.115847 36403558 PMC9625846

[28] Esplund JN, Taylor AD, Stone TJ, Palavecino EL, Kilic A, Luther VP, Ohl CA, Beardsley JR. 2023. Clinical impact of a multiplex rapid diagnostic pneumonia panel in critically ill patients. Antimicrob Steward Healthc Epidemiol 3:e5. doi:10.1017/ash.2022.358 36714280 PMC9879924

[29] Gadsby NJ, Russell CD, McHugh MP, Mark H, Conway Morris A, Laurenson IF, Hill AT, Templeton KE. 2016. Comprehensive molecular testing for respiratory pathogens in community-acquired pneumonia. Clin Infect Dis 62:817–823. doi:10.1093/cid/civ1214 26747825 PMC4787606

[30] Sangil A, Calbo E, Robles A, Benet S, Viladot ME, Pascual V, Cuchí E, Pérez J, Barreiro B, Sánchez B, Torres J, Canales L, De Marcos JA, Garau J. 2012. Aetiology of community-acquired pneumonia among adults in an H1N1 pandemic year: the role of respiratory viruses. Eur J Clin Microbiol Infect Dis 31:2765–2772. doi:10.1007/s10096-012-1626-6 22549730 PMC7088264

[31] Rello J, Ulldemolins M, Lisboa T, Koulenti D, Mañez R, Martin-Loeches I, De Waele JJ, Putensen C, Guven M, Deja M, Diaz E, EU-VAP/CAP Study Group. 2011. Determinants of prescription and choice of empirical therapy for hospital-acquired and ventilator-associated pneumonia. Eur Respir J 37:1332–1339. doi:10.1183/09031936.00093010 20847075

[32] Welte T. 2016. Severe pneumonia in the intensive care unit. Med Klin Intensivmed Notfmed 111:279–289. doi:10.1007/s00063-016-0165-9 27160261

[33] Holter JC, Müller F, Bjørang O, Samdal HH, Marthinsen JB, Jenum PA, Ueland T, Frøland SS, Aukrust P, Husebye E, Heggelund L. 2015. Etiology of community-acquired pneumonia and diagnostic yields of microbiological methods: a 3-year prospective study in Norway. BMC Infect Dis 15:64. doi:10.1186/s12879-015-0803-5 25887603 PMC4334764

[34] Howard LSGE, Sillis M, Pasteur MC, Kamath AV, Harrison BDW. 2005. Microbiological profile of community-acquired pneumonia in adults over the last 20 years. J Infect 50:107–113. doi:10.1016/j.jinf.2004.05.003 15667910

[35] Drijkoningen JJC, Rohde GGU. 2014. Pneumococcal infection in adults: burden of disease. Clin Microbiol Infect 20 Suppl 5:45–51. doi:10.1111/1469-0691.12461 24313448

[36] Cvitkovic Spik V, Beovic B, Pokorn M, Drole Torkar A, Vidmar D, Papst L, Seme K, Kogoj R, Müller Premru M. 2013. Improvement of pneumococcal pneumonia diagnostics by the use of rt-PCR on plasma and respiratory samples. Scand J Infect Dis 45:731–737. doi:10.3109/00365548.2013.804631 23826792

[37] Kogoj R, Praprotnik M, Mrvič T, Korva M, Keše D. 2018. Genetic diversity and macrolide resistance of Mycoplasma pneumoniae isolates from two consecutive epidemics in Slovenia. Eur J Clin Microbiol Infect Dis 37:99–107. doi:10.1007/s10096-017-3106-5 28948376

[38] Gramegna A, Sotgiu G, Di Pasquale M, Radovanovic D, Terraneo S, Reyes LF, Vendrell E, Neves J, Menzella F, Blasi F, Aliberti S, Restrepo MI, GLIMP Study Group. 2018. Atypical pathogens in hospitalized patients with community-acquired pneumonia: a worldwide perspective. BMC Infect Dis 18:677. doi:10.1186/s12879-018-3565-z 30563504 PMC6299604

[39] Waites KB, Xiao L, Liu Y, Balish MF, Atkinson TP. 2017. Mycoplasma pneumoniae from the respiratory tract and beyond. Clin Microbiol Rev 30:747–809. doi:10.1128/CMR.00114-16 28539503 PMC5475226

[40] Ruuskanen O, Järvinen A. 2014. What is the real role of respiratory viruses in severe community-acquired pneumonia? Clin Infect Dis 59:71–73. doi:10.1093/cid/ciu242 24729504 PMC4305147

[41] Quinton LJ, Walkey AJ, Mizgerd JP. 2018. Integrative physiology of pneumonia. Physiol Rev 98:1417–1464. doi:10.1152/physrev.00032.2017 29767563 PMC6088146

[42] Enne VI, Aydin A, Baldan R, Owen DR, Richardson H, Ricciardi F, Russell C, Nomamiukor-Ikeji BO, Swart A-M, High J, Colles A, Barber J, Gant V, Livermore DM, O’Grady J. 2022. Multicentre evaluation of two multiplex PCR platforms for the rapid microbiological investigation of nosocomial pneumonia in UK ICUs: the INHALE WP1 study. Thorax 77:1220–1228. doi:10.1136/thoraxjnl-2021-216990 35027473

[43] Tellapragada C, Giske CG. 2021. The Unyvero Hospital-acquired pneumonia panel for diagnosis of secondary bacterial pneumonia in COVID-19 patients. Eur J Clin Microbiol Infect Dis 40:2479–2485. doi:10.1007/s10096-021-04194-6 33661410 PMC7930892

[44] Andrews V, Pinholt M, Schneider UV, Schønning K, Søes LM, Lisby G. 2022. Performance of PCR-based syndromic testing compared to bacterial culture in patients with suspected pneumonia applying microscopy for quality assessment. APMIS 130:417–426. doi:10.1111/apm.13232 35499302

[45] Søgaard KK, Hinic V, Goldenberger D, Gensch A, Schweitzer M, Bättig V, Siegemund M, Bassetti S, Bingisser R, Tamm M, Battegay M, Weisser M, Stolz D, Khanna N, Egli A. 2024. Evaluation of the clinical relevance of the Biofire FilmArray pneumonia panel among hospitalized patients. Infection 52:173–181. doi:10.1007/s15010-023-02080-1 37572241 PMC10810975

[46] Wu X, Li Y, Zhang M, Li M, Zhang R, Lu X, Gao W, Li Q, Xia Y, Pan P, Li Q. 2020. Etiology of severe community-acquired pneumonia in adults based on metagenomic next-generation sequencing: a prospective multicenter study. Infect Dis Ther 9:1003–1015. doi:10.1007/s40121-020-00353-y 33170499 PMC7652912

