# Supplementary material for: Syndromic molecular testing in mechanically ventilated patients with severe pneumonia: a supportive diagnostic approach
Source: Microbiol Spectr. 2025 Nov 21;14(1):e02155-25. doi: 10.1128/spectrum.02155-25 (PMC12772339; doi:10.1128/spectrum.02155-25)
Supplement: Table S1 — Ct values for S. pneumoniae and H. influenzae upon ADA. [file spectrum.02155-25-s0001.docx]

| Table S1. Ct values of ADA-positive *S. pneumoniae* and *H. influenzae* samples according to CDA results. | | |
| --- | --- | --- |
| ***S. pneumoniae*** | | |
| ADA pos / CDA pos (Ct values) | ADA pos /CDA neg (Ct values) | *t-test* |
| 23.01 | 31.85 | *p* = 0.001 |
| 22.21 | 26.62 |  |
| 29.89 | 29.46 |  |
| 21.45 | 32.4 |  |
| 20.36 | 28.93 |  |
| 24.64 | 24.22 |  |
| 22.21 | 28.62 |  |
| 17.97 |  |  |
| 20.56 |  |  |
| 25.77 |  |  |
| ***H. influenzae*** | | |
| ADA pos / CDA pos (Ct values) | ADA pos /CDA neg (Ct values) | *t-test* |
| 24.61 | 23.76 | *p* < 0.001 |
| 22.81 | 31.97 |  |
| 16.98 | 30.13 |  |
| 21.25 | 32.6 |  |
| 19.59 | 32.23 |  |
| 24.82 | 32.5 |  |
| 23.76 |  |  |
| ADA = Allplex molecular diagnostic approach; CDA = Conventional diagnostic approach | | |
